# The NIDDK Information Network: A Community Portal for Finding Data, Materials, and Tools for Researchers Studying Diabetes, Digestive, and Kidney Diseases

**DOI:** 10.1371/journal.pone.0136206

**Published:** 2015-09-22

**Authors:** Patricia L. Whetzel, Jeffrey S. Grethe, Davis E. Banks, Maryann E. Martone

**Affiliations:** 1 Center for Research in Biological Systems, University of California, San Diego, San Diego, California, United States of America; 2 Dept of Neurosciences, University of California, San Diego, San Diego, California, United States of America; University of Connecticut, UNITED STATES

## Abstract

The NIDDK Information Network (dkNET; http://dknet.org) was launched to serve the needs of basic and clinical investigators in metabolic, digestive and kidney disease by facilitating access to research resources that advance the mission of the **National Institute of Diabetes and Digestive and Kidney Diseases (NIDDK)**. By research resources, we mean the multitude of data, software tools, materials, services, projects and organizations available to researchers in the public domain. Most of these are accessed via web-accessible databases or web portals, each developed, designed and maintained by numerous different projects, organizations and individuals. While many of the large government funded databases, maintained by agencies such as European Bioinformatics Institute and the National Center for Biotechnology Information, are well known to researchers, many more that have been developed by and for the biomedical research community are unknown or underutilized. At least part of the problem is the nature of dynamic databases, which are considered part of the “hidden” web, that is, content that is not easily accessed by search engines. dkNET was created specifically to address the challenge of connecting researchers to research resources via these types of community databases and web portals. dkNET functions as a “search engine for data”, searching across millions of database records contained in hundreds of biomedical databases developed and maintained by independent projects around the world. A primary focus of dkNET are centers and projects specifically created to provide high quality data and resources to NIDDK researchers. Through the novel data ingest process used in dkNET, additional data sources can easily be incorporated, allowing it to scale with the growth of digital data and the needs of the dkNET community. Here, we provide an overview of the dkNET portal and its functions. We show how dkNET can be used to address a variety of use cases that involve searching for research resources.

## Introduction

Modern biomedical science is increasingly a digital enterprise, involving the accrual of ever larger numbers and types of digital assets. Such assets include not only the digital forms of narrative works like articles and books, but increasingly the software, code, data, and workflows that underpin biomedical science. Despite the increasing amount of data and tools available in the public domain, discovering, accessing and utilizing these considerable resources remains difficult. These difficulties have led major funding agencies such as the US National Institutes of Health to consider new types of services around data and software discovery [1–2]. Compared to search, indexing and citation systems for journals and books, we are still in the very early days of developing systems and standards for important assets like research data. Therefore, in the age of “Big Data”, calls to add to the growing amount of digital assets in the public domain must also be accompanied by platforms for making them discoverable and thereby re-usable by the community, much in the way that PubMed allows collective search across the breadth of biomedical literature [1].

The NIDDK Information Network (dkNET; http://dknet.org) was launched to serve the needs of basic and clinical investigators in metabolic, digestive and kidney disease by providing seamless access to large pools of research resources, relevant to the mission of the National Institute of Diabetes and Digestive and Kidney Diseases (NIDDK). By research resources, we mean the multitude of data, software tools, materials, services, projects and organizations available to researchers in the public domain. Access to these research resources is generally provided by a database or web portal, each maintained by a different organization, project or individual. However, while many of the large government funded databases, maintained by agencies such as European Bioinformatics Institute and the National Center for Biotechnology Information, are well known to researchers, many more that have been developed by and for the biomedical research community are unknown or underutilized [3]. Despite the availability of search engines such as Google, and focused efforts such as the *Nucleic Acids Research* Database issue, searching, accessing and utilizing these thousands of data resources remains challenging and inefficient. At least part of the problem is the nature of dynamic databases, which are considered part of the “hidden” web, that is, content that is not easily accessed by search engines [4].

dkNET was created specifically to address the challenge of connecting researchers in metabolic and digestive diseases to research resources via these types of community databases and web portals. dkNET functions as a “search engine for data”, searching across millions of database records contained in hundreds of biomedical databases developed and maintained by independent projects around the world. Through the novel data ingest process used in dkNET, additional data sources can easily be incorporated, allowing it to scale with the growth of digital data and the needs of the dkNET community. dkNET is the successor to dkCOIN, a pilot project to test the feasibility of connecting a small set of NIDDK-funded resources within a unified framework [5].

dkNET was launched in April 2014. It is built upon a unique data platform, SciCrunch, developed originally by the Neuroscience Information Framework (NIF, [3, 6–8]). The NIF project was initiated in 2006 with aims similar to dkNET: to create a repository of neuroscience resources to connect researchers to the many research resources being produced by NIH and elsewhere for their benefit [9]. To achieve these goals, NIF developed an architecture and set of tools for indexing the contents of individual databases for search [6–7, 10]. Although the focus of the NIF is neuroscience, the data architecture itself is generic and applicable as a general search engine for data. The SciCrunch infrastructure allows communities like dkNET to create domain-specific data portals on top of a set of shared data resources. A goal of SciCrunch is to ensure that different communities can leverage efforts across biomedicine, through the creation of networked data resources, so as to continually add to the amount of data and resources that are discoverable and usable. Thus, although dkNET is a customized portal built for the metabolic, digestive and kidney disease community, it draws from and adds to a growing biomedical data discovery index.

In the following, we provide an introduction to the dkNET data portal, describing the architecture, organization and functionality. We then illustrate the utility of dkNET by providing a set of use cases of relevance to NIDDK researchers.

## Materials and Methods

### SciCrunch

dkNET is developed within the SciCrunch (http://scicrunch.org) infrastructure. SciCrunch employs a lightweight strategy to data federation, using Lucene/Solr to create a semantically enhanced meta-index over the deeper content of digital databases. The basic SciCrunch infrastructure provides for a data ingest pipeline, described in the next section, a resource management dashboard, and a search portal that utilizes ontologies to provide enhanced keyword search. Basic data display, analytics and export functions are also provided. SciCrunch provides a PHP based web interface and REST Web services in conjunction with the data ingest pipeline tools and ontology store [6,10]. dkNET 2.0, released in April 2015, included a re-design utilizing the open source bootstrap framework (http://getbootstrap.com) so that the site is fully responsive and is usable on mobile and tablet devices.

SciCrunch uses the NIF Standardized Ontologies (NIFSTD; [8, 11]) to enhance search. Although originally developed for neuroscience, NIFSTD actually is a set of modular ontologies, some of which were developed by the broader community as part of the OBO ontologies [12]. As additional community ontologies have been developed and matured, NIFSTD gradually replaced custom neuroscience content with ontologies that provide more general coverage of biomedical systems, e.g., the Gene Ontology and UBERON [13], making them generally applicable to multiple domains. These ontologies are used to provide search expansions, e.g., synonyms, acronyms and related classes and provide the semantic framework for organizing data sources within SciCrunch portals (see below).

### Data ingest

Data sources for inclusion into SciCrunch undergo a two-tier registration process. First, a high level description of the data source is created in the SciCrunch Registry. This initial step collects metadata about the resource such as the name, description, URL and type of data provided by the database. This step is accomplished through a form-based interface and may be performed by non-programmers. Some SciCrunch communities, e.g., NIF, allow the community at large to recommend a resource for inclusion. In dkNET, however, all registration is currently performed by community curators.

Deeper registration of resource content into the SciCrunch Data Federation is accomplished through DISCO [7, 10]. As data ingest requires some programming and curation skills, it is generally performed by a skilled community curator. Curators create a machine-readable description of the data through the creation of an interop(erability) file that specifies how data are to be structured and ingested. DISCO currently ingests data provided via web services, databases, or in documents. SciCrunch employs a custom Concept Mapper tool that provides a curatorial interface for annotating data and determining functions such as facets, display and weighting of columns. SciCrunch performs a light-weight semantic alignment of data sources through mapping columns to one or more domains of our upper ontology. Mapping columns helps users restrict their search to certain types of entities, e.g., diseases, organisms, anatomical structures and cells, although it is not required as part of the data curation process. These mappings are used to apply specialized keyword filters to the data via a custom query syntax.

### The dkNET portal

**Fig 1:**
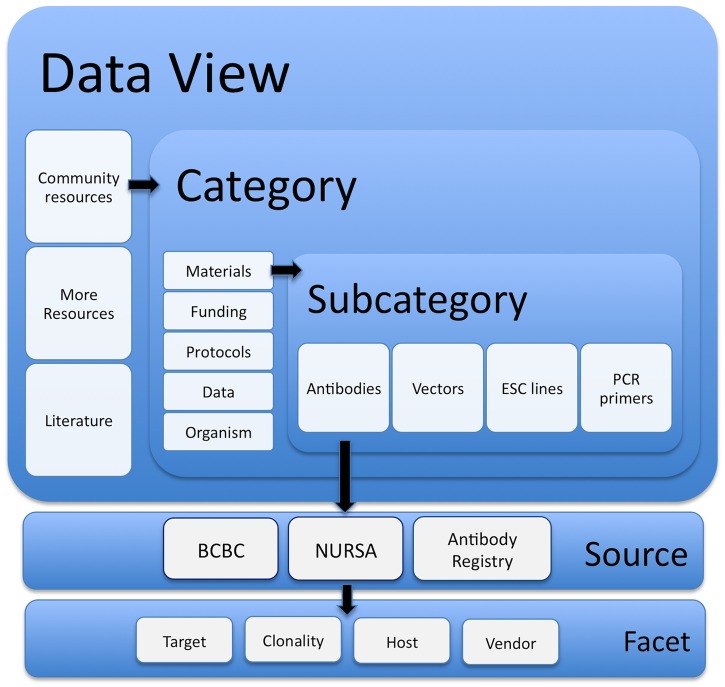
Schematic overview of dkNET Navigation that illustrates the progressive refinement of information within the system. At the top level, dkNET organizes resources under 3 views: Community Resources, More Resources and Literature. Within each view are specific categories, e.g., for Community Resources, the categories comprise Materials, Funding, Protocols, Data and Organism. Each of these, in turn, may be expanded into several subcategories, e.g., Materials is subdivided into Antibodies, Vectors, Embryonic Stem Cell (ESC) lines and PCR primers. Once a Subcategory is selected, a set of relevant sources are provided, each with a set of appropriate facets. Not all levels may be available for all subsections, e.g., a category may not have subcategories or a given source may not have facets.

Setting up a resource portal using SciCrunch involves selecting which data sources are to be made available and how they are to be organized. For dkNET, we ingested 6 new data sources supported wholly or in part by NIDDK (Table 1). A given source may be broken up into multiple views by use of appropriate filters, and so may appear under multiple categories. Additional relevant sources were selected from those available through the SciCrunch Data Federation. The community curator decides how the data are to be organized within the portal through the selection of Data Views and the specification of Categories and Subcategories. The initial specification of these categories and subcategories followed the organization established by dkCOIN. The basic navigation scheme used by dkNET is shown in Fig 1.

**Table 1:** List of resources currently available as Community Resources and abbreviations used in text.

Resource	Abbreviation	Description
AddGene		A repository of plasmids for use in research.
AntibodyRegistry		An authoritative source of antibody identifiers, which can be used to uniquely reference antibodies in publications.
Beta Cell Biology Consortium*	BCBC	A consortium of researchers working to advance the understanding of pancreatic islet development and function. The BCBC provides access to adenoviruses, antibodies, genomic studies, mouse embryonic stem cell lines, mouse models, and protocols.
Diabetic Complications Consortium*	DiaComp	A consortium of researchers involved in complications research related to diabetes. Resources provided include animal models, assays, histology data, and protocols.
GenitoUrinary Molecular Anatomy Project*	GUDMAP	A consortium of laboratories that provide a molecular atlas of gene expression for the developing organs of the GenitoUrinary tract.
Grants.gov		Provides information on over 1,000 grant programs open for application.
Integrated Animals		A virtual database containing animal models from the Zebrafish International Resource Center, Rat Genome Database, Caenorhabditis Genetics Center, International Mouse Strain Resource, Mouse Genome Informatics, the Zebrafish Model Organism Database, and Bloomington Drosophila Stock Center.
Integrated Grants		A virtual database listing funded research resources including NIH Research Portfolio Online Reporting Tool.
National Mouse Molecular Phenotyping Centers*	MMPC	Provides metabolic and physiologic phenotyping services for mouse models of diabetes, diabetic complications, obesity and related disorders.
Nuclear Receptor Signaling Atlas*	NURSA	Provides information that advances understanding of the structure, function, and role in disease of nuclear receptors (NRs) and coregulators.
T1Dbase*		Provides annotated genomic sequences for suspected T1D susceptibility regions; genetic data; microarray data; and "global" datasets, generally from the literature.

* Resources funded wholly or in part by NIDDK.

## Results

### Design of dkNET

dkNET comprises a community-based informational website and the resource search portal. The community website provides basic information about the dkNET project such as goals, project members, training information and community news and list of events, e.g., webinars, NIH training opportunities, etc. The resource search portal provides access to the data federation and search functions. With the launch of dkNET 2.0, the main website will be phased out, as its content has been moved to the search portal. In this article, we focus our description on the search portal. The current screenshots and query results represent the state of dkNET in June 2015. As the search portal is constantly undergoing improvements and the content continually changes, the interface and results sets may change over time.

dkNET was designed for publicly available data. Use of the portal requires no account for basic use. Customized functions, e.g., saved searches, require a simple user registration and log in, available in the upper right corner of the portal. Both the use of the portal and user registration are free. In some cases, however, download of the data may be restricted, depending upon the licenses applied by the source. A set of tutorials and other help functions are provided under the ABOUT section on the portal main page.

### dkNET navigation

**Fig 2:**
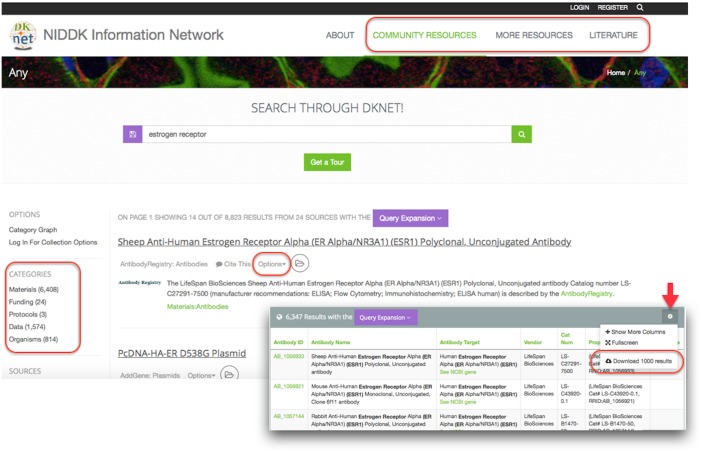
Main results page of dkNET search portal as of June, 2015. Search results are organized within 3 data views, indicated by the categories in the upper right menu (rectangle), and also via categories (rectangle, left side), subcategories and sources (left navigation menu). Results can be displayed as snippets (main window) or as a table (lower right) by selecting the Options menu next to each result. Results sets may be saved (see text) or exported as CSV files by selecting “Download first 1000 results” (oval) accessed via the tool icon in the upper right corner of the table view (red arrow).

As described in the introduction, dkNET draws upon the hundreds of biomedical databases spanning multiple data types, organisms, and biological systems made available via SciCrunch. The central challenge for effective utilization of these resources is developing an organizational framework and search strategy that makes it easy for a user to understand what is available and how to find resources of relevance to their research. With hundreds of databases comprising millions of records, it is easy for a user to get overwhelmed with the amount of data available. To accommodate the need to promote awareness and use of dkNET-specific resources while at the same time providing broader search when required, the search portal was divided into 3 major Views, each represented by a separate tab: Community Resources, More Resources and Literature (Fig 2). As each tab is selected, the same search is executed across the different views.

#### Community resources

The Community Resources Data View is the section that provides a given community the opportunity to select certain data sources and organize them according to their needs. In the case of dkNET, Community Resources provides access to a limited number of databases (Table 1) that were specifically developed for NIDDK-supported researchers and ingested via dkNET or were selected from the SciCrunch data federation because they were viewed as specifically valuable to the dkNET community. An example of the latter is the Antibody Registry, a database of over 2 million antibodies for biomedical science developed by the NIF project. Within the Community Resources section, results are further organized according to several high level categories, currently: Materials, Funding, Data, Protocols, and Organisms. These categories, in turn, may be broken into several relevant sub-categories, e.g., the category Materials is divided into antibodies, vectors, embryonic stem cells, and PCR primers (Figs 1 and 2). The initial set of subcategories was chosen by the community curator based on the organization of resources within the original dkCOIN portal, but has been supplemented with new categories as new data have been ingested, e.g., expression data via GUDMAP and gene variants from T1DBase. Resource abbreviations used in text are listed in Table 1.

#### More resources

**Fig 3:**
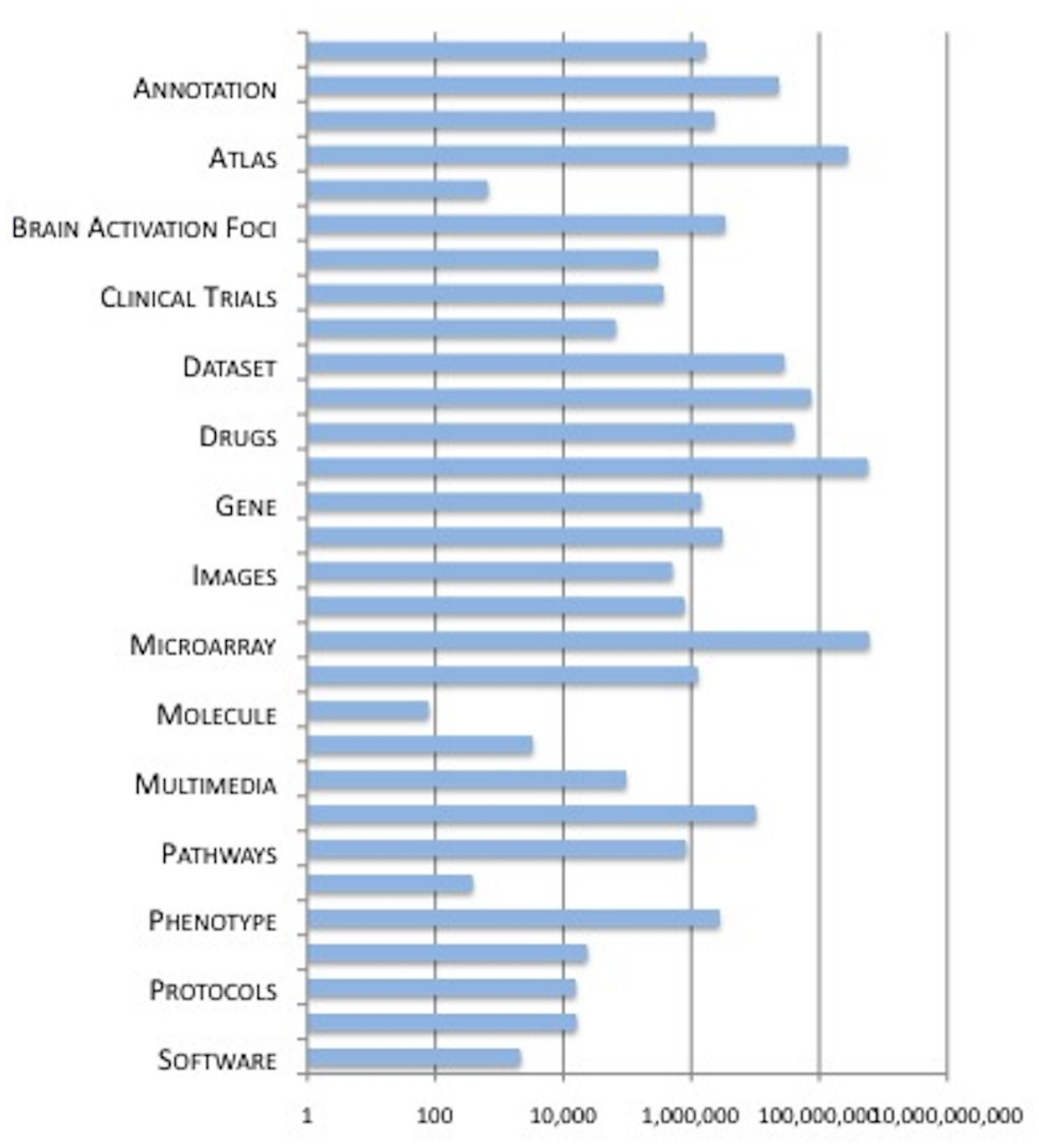
Summary of the current coverage of the data content available through the dkNET More Resources tab (Oct, 2014). The major categories are displayed on the Y axis, while the number of records for each category of data is displayed on the X axis. For legibility, only some of the categories are shown.

The More Resources section provides access to the 200+ databases available through SciCrunch. SciCrunch constantly adds new data sources, as each SciCrunch community builds their federated data sources. When a data source is ingested, it is categorized by the SciCrunch curation team according to the system level that characterizes the data, e.g. gross anatomy, cell, molecule, and the main type of resource it offers, e.g., animal, cell line, or type of content, e.g., images. Unlike the community section, the categories and facets are not configurable by an individual community. An overview of the content and coverage of More Resources is shown in Fig 3. An individual data source may be represented by multiple views, which may appear under different categories. SciCrunch also contains several integrated views, created through alignment of multiple sources covering the same type of data. In this case, the curators will select a set of common fields and align the data across each source. These views are identified by the prefix “Integrated” followed by the data category, e.g., Integrated: Software.

Results from a query can be further refined by using the categories in the left navigation menu. Categories include both broad data types, e.g., expression, images, and system level, e.g., gross anatomy, cell. Results are returned according to a relevancy ranking that is determined by the number of times the search terms appear within a source, multiplied by a weighting factor applied to certain columns during the data curation process. For example, columns containing entities mapped to one of our main ontology categories, e.g., gene, cell, anatomical structure, are weighted more heavily than results from columns containing free-text descriptions or references. Selecting a source will provide a tabular view of results and a set of custom facets for that source. More details on the use of different search features and filters will be provided in the Use Case section below.

Use of each data source by other communities in SciCrunch is indicated by colored circle with a number next to each source. The higher the number, the more communities using a particular data source. Users can sort data sources based on usage by individual communities in the side navigation menu. In this way, dkNET users can find sets of data that are deemed important by other communities of expertise, e.g., neuroscience or aging, and take advantage of any views that these communities apply to data sources within their domain of expertise.

#### Literature

Despite the hundreds of databases available for search, most research resources are only described within the literature. Thus, to make it easier for researchers to navigate between databases and the literature, dkNET also provides simultaneous search across the scientific literature from PubMed and PubMedCentral. The literature data results can be filtered by author, year, and journal, although unlike PubMed, users cannot currently restrict searches to these categories. Users can also execute their searches across the full text articles from the open access subset of PubMedCentral by selecting the “full text” facet. dkNET also allows the user to restrict their search to particular sections of the paper, e.g., methods, results.

dkNET has incorporated Altmetrics (http://www.altmetric.com/) into the literature results. Altmetrics is a new measure for analyzing the impact of scholarly research based on mentions in social media, traditional media, and online reference managers [14]. When they are available, an Altmetric icon is displayed next to the article. As research findings are increasingly posted to the web in a data repository, blog, or mentioned in a Tweet, the Altmetric service can point readers to interesting discussions around a particular article.

### Search of dkNET

**Fig 4:**
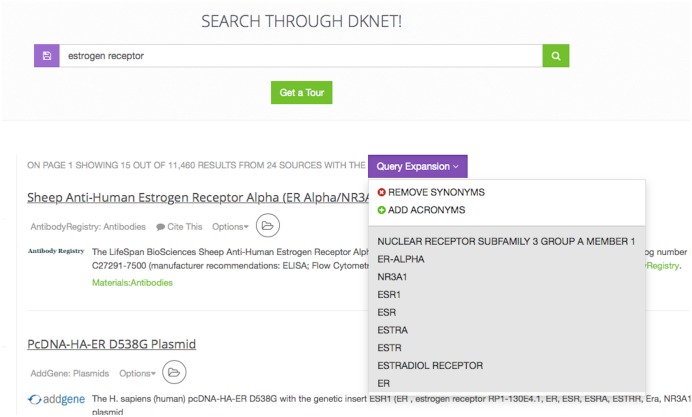
Query expansion. The included terms are additional terms used to query the data sources based on the term expansion process using knowledge from the ontology. The most common expansion includes synonyms and common abbreviations of a term.

dkNET provides an enhanced key word search interface. Users enter terms into the search box (Fig 2), and search is executed across all three Views of dkNET. As users type in terms, they are provided with a set of options to autocomplete their search term based on the dkNET vocabularies. If the search term or phrase is available, it is automatically expanded to include synonyms and common abbreviations of a term. For example the search term estrogen receptor is expanded to nuclear receptor subfamily 3 group a member 1, er-alpha, nr3a1, esr1, esr, estra, estr, estradiol receptor, er and all terms are used when searching through data in dkNET (Fig 4).

For certain types of entities, the expansion also includes additional related entities, e.g., specific cell types, or related techniques. These expansions are based on the underlying structure of the ontology where an equivalency statement has been included. The majority of these come via the NIF project, e.g., a hippocampal neuron is equivalent to all classes of neurons that are found in the hippocampus [8]. Future versions of dkNET will include similar equivalency classes for entities specifically relevant to the kidney, digestive and genitourinary systems.

In many cases, the expanded search helps to identify records that would be missed with just a simple key word search. For example, 8,823 results are returned when using the expansion with the search term estrogen receptor while only 2400 results are returned without the expansion. However, in other cases, particularly where a synonym is used in multiple contexts, the expansion may lead to many false positives. Thus, the user has the option to disable the term expansion by clicking on the “Remove synonyms” and therefore reducing the number of results. Although dkNET uses the ontology to enhance search, search is not constrained by the ontology, that is, dkNET still executes the search as a string with no expansions.

dkNET provides for basic boolean operators to refine search: AND, OR and NOT. The default operator is AND, i.e., compound search terms are joined by an AND, for terms entered into the search box. The AND operator currently operates per record and not per data source; that is, for a compound query, e.g., diabetes AND obesity, sources will be returned only if they contain individual records with both search terms. NOT queries may be performed by placing a hyphen before the term to be negated. Negation is useful for helping to narrow down search results, e.g., searching for: diabetes-digestive, will remove results that contain the text ‘National Institute of Diabetes, Digestive and Kidney diseases’.

dkNET provides the capacity to restrict searches to certain columns contained within the source by using a special syntax. Entering Anatomy:kidney will search for the term kidney only in columns that have been mapped to the high level concept Anatomy during the curation process. This type of search restriction is very useful for reducing the numbers of false positives returned, for example, when the same term is found in multiple types of entities. A variant on the restricted search is the “find” command, which looks for data sources that have columns containing certain types of entities, e.g., find:gene kidney, will look for all data sources that have the term kidney *and* a column mapped to gene.

The parameters of a search can be saved in order to re-run a search at a later time by selecting the “Save search” icon next to the search bar. The saved parameters include the search term(s), category and subcategories, and resource being viewed at the time the search is saved. Users must have an account and be logged in to use this feature.

### Display

dkNET provides a variety of options and tools for displaying, exploring, refining and exporting search results. Because the Community Resources section has been customized for dkNET based on user-testing and community feedback, we focus on the special features available for display and browsing in this section. Use of display features in the More Resources section are presented in the use cases section below.

dkNET has adopted several conventions from existing search engines to make the display of results derived from these different databases unified and easy to browse. Search results are presented as a list of relevant records found in the different resources included in the Community Resources section. Each individual record is characterized by a snippet of text that is defined by the community curator and automatically constructed from the main attributes of the particular data source. Users can quickly browse through the list, much as one does a set of web page results to quickly understand the sources and nature of the data. If results are returned from more than one source, the snippets from individual sources are interleaved to allow users to quickly determine what types of data are available for each source. More details about each source are provided by clicking on “View source information” under options (Fig 2), whereas more details about each record can be viewed by clicking on the title for that record. Users can sort results according to category or subcategory or source by using the left navigation menu.

Users are presented with several options for changing the results display. Selecting the “Table view” under the options menu (Fig 2) will display more details about results from a resource. This includes facets, which can be used to further narrow a search as described below. Table views may be exported as spreadsheets in CSV format by selecting the “Dpwnload 1000 results” button in the upper right corner of the display window (Fig 2). As indicated, manual download is restricted to 1000 results. The entire result set can be accessed programmatically by obtaining an API key from dkNET.

As described above for the More Resources section, results are ranked according to a general ranking algorithm that takes into account mentions of the search term weighted according to the type of column in which it appears. For some sources with dated entries, this ranking strategy may not be optimal, as it does not currently order the results according to date. However, using the in column functions, users can easily sort the results according to any column, as described in the next section.

### Faceted search and filtering

**Fig 5:**
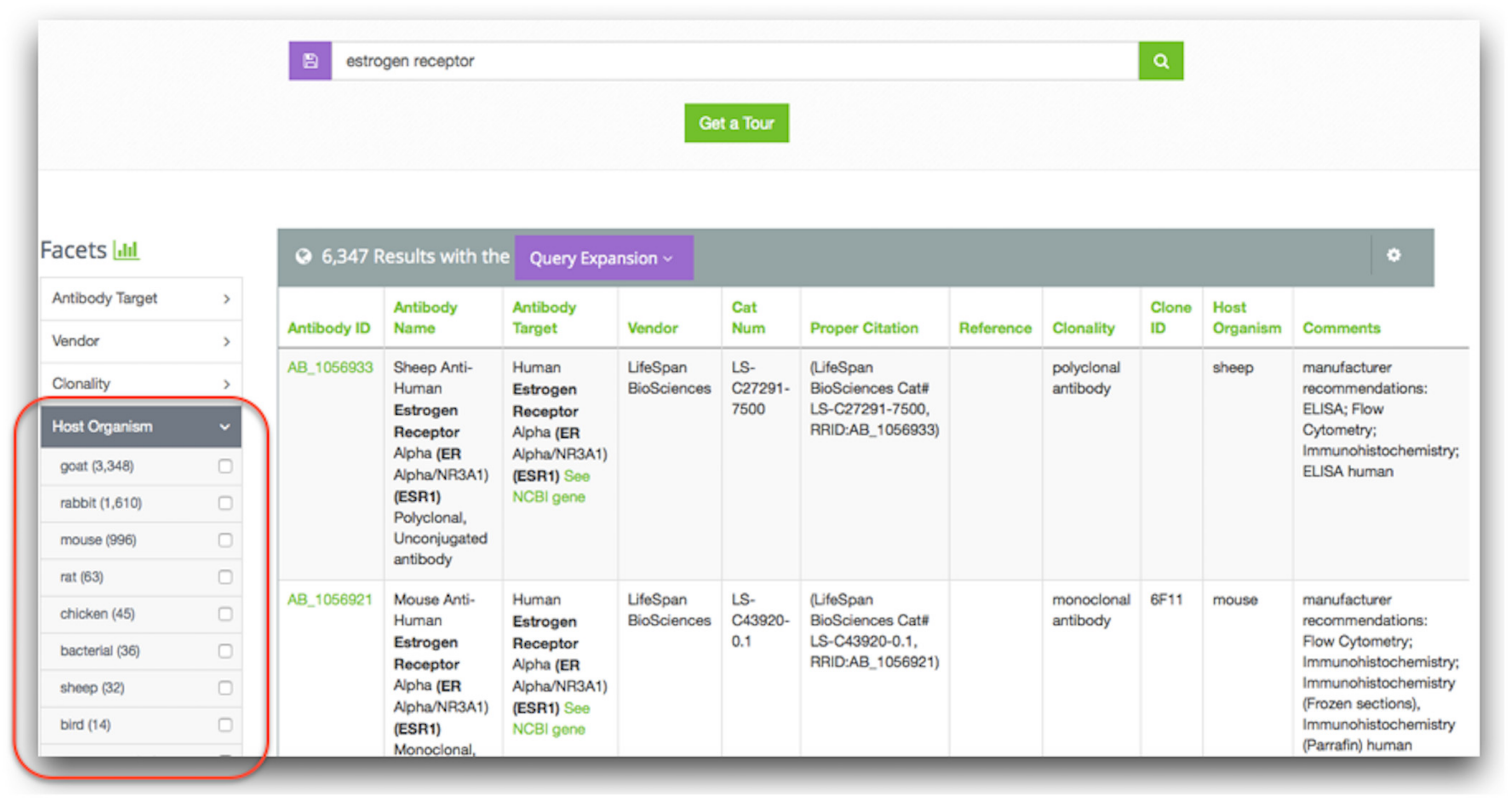
Example of faceted data in dkNET. Data columns that contain repeated values are selected by the data curators to be faceted. This is one type of ‘filter’ to help users narrow search results.

Within the search results from the Community Resources section, the results can be further refined using facets and filters. The facets are determined at the time of data curation and are selected based on input data columns that contain values that are frequently repeated in the column. For example, it is useful to add a facet to a column that contains gene names or anatomical locations, which are often repeated in a data column. By adding a facet, it is easy to see all possible values of data in that column and the total count of occurrences of the value for a given resource (Fig 5). Once a facet is selected, the total number of data results is limited to only those rows that contain the selected value. Data can be faceted by more than one value within a column or by more than one column. Refinement of search results is not limited to only known, repeated values within a column. Each column can also be filtered by any text provided by the user by clicking on the column header and entering text of interest (see Use Case 1 below). dkNET provides a category analytics tool that provides an overview of the content of a given data category or data set. This view can be created by clicking on the icon in the left navigation menu.

### Collections and saved searches

dkNET provides the ability to save individual results to a collection, similar to the shopping cart function in most e-retail sites, and to save searches. However, unlike typical shopping carts, users may create multiple collections, which are saved for future sessions. To access these functions, users must sign up for an account and be logged in.

**Fig 6:**
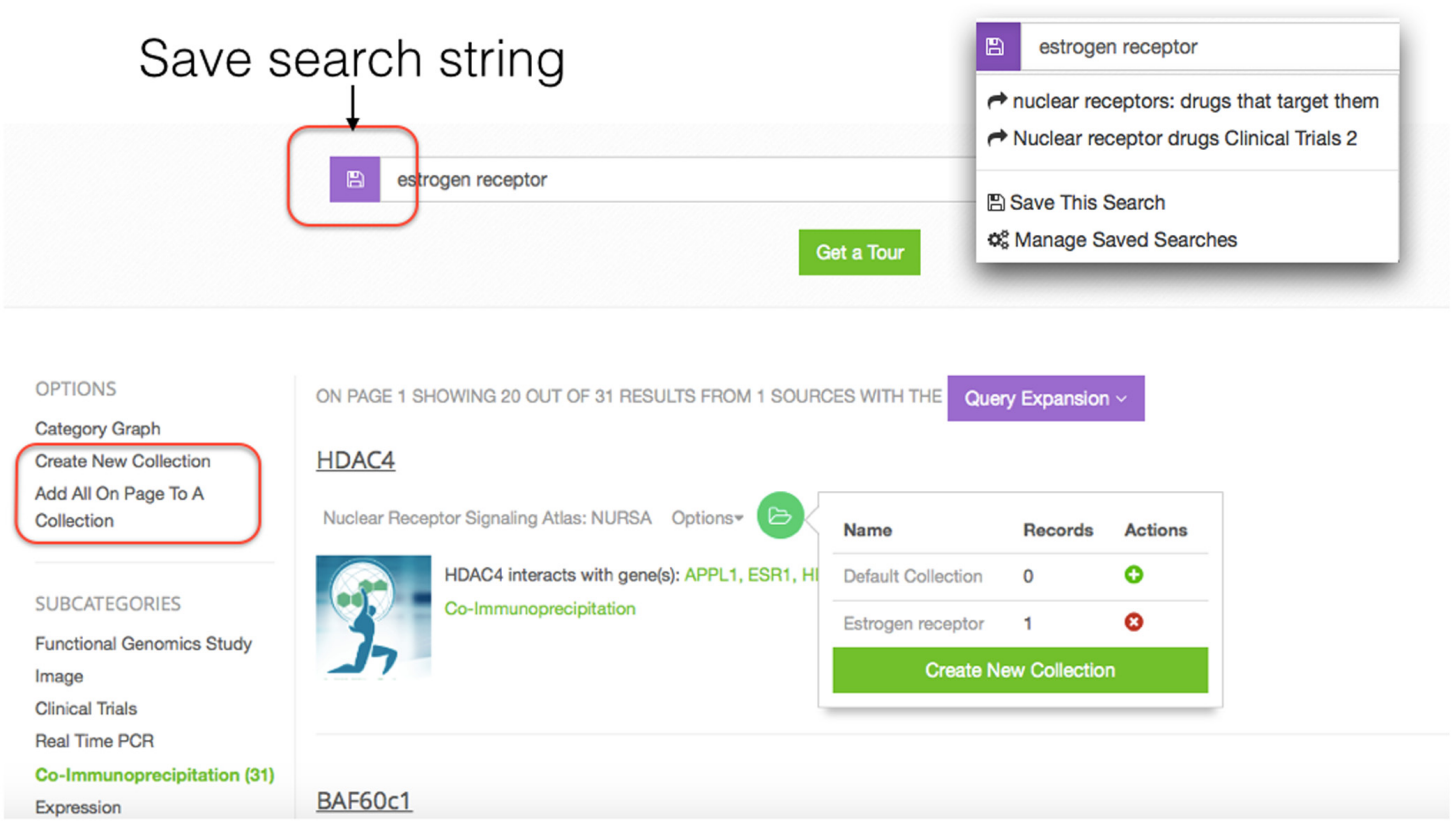
Collections and Save Searches Users can create collections to store data records. A record can be added to one or more collections. All records on a page can be added to a collection by selecting the “Add all records…” option in the left navigation menu. Searches are saved by selecting the icon to the left of the search bar. A menu is then displayed (inset, upper right) that shows previously saved searches, along with functions to save the current search.

The Collections function is currently only available for the snippet view in the Community Resource section. All users are assigned a default collection to store records and can also create new collections. A record can be added to a collection individually by clicking on the “Add to Collections” icon displayed for each result record or all records on a page can be added to a collection using the “Add all records” button (Fig 6). The list of all collections is visible from the user account for “My Collections” and from here the contents of the collection can be reviewed and managed via operations such as re-name, export, or delete.

Searches may be saved by clicking on the “Save search” icon to the left of the search bar (Fig 6). The same icon will show previously saved searches (insert, upper right).

### Use cases

To illustrate the use of dkNET and the types of questions that can be addressed, we provide a series of use cases, originally supplied by members of the dkNET Advisory Board and other members of the community. For each, we show sample queries and search strategies that can be used to retrieve relevant results. As with any search system that supports discovery, there are different strategies and approaches that can be used to address any given use case. In each case, we assume that a user will be starting at the main search page unless otherwise indicated. The result sets represent the state of dkNET in June 2015. As dkNET data sources are dynamically updated and new sources are added on a regular basis, the same query may not give the same results at different times.

#### [UC1] Find funding opportunities for drosophila and diabetes

Query: drosophila diabetes funding opportunities. Navigation: Community Resources > Funding > Funded Grants > NIH Reporter. Execution of the above query takes the user to results under the Funding category. dkNET provides access to two sources of funding information: funding opportunities and funded grants. These two types are listed in the subcategories in the left navigation menu. Below the subcategories are listed the sources for each. For funding opportunities, we access Grants.gov, which combines program announcements from government agencies in the United States. For awarded grants, we access an Integrated Source, comprising mainly the NIH RePORTER (http://projectreporter.nih.gov/), maintained by the US National Institutes of Health, but supplemented with some additional databases that provide information on funded awards outside of NIH. In this case, results were only returned under Funded Grants, indicating that the search terms were not found within Grants.gov.

**Fig 7:**
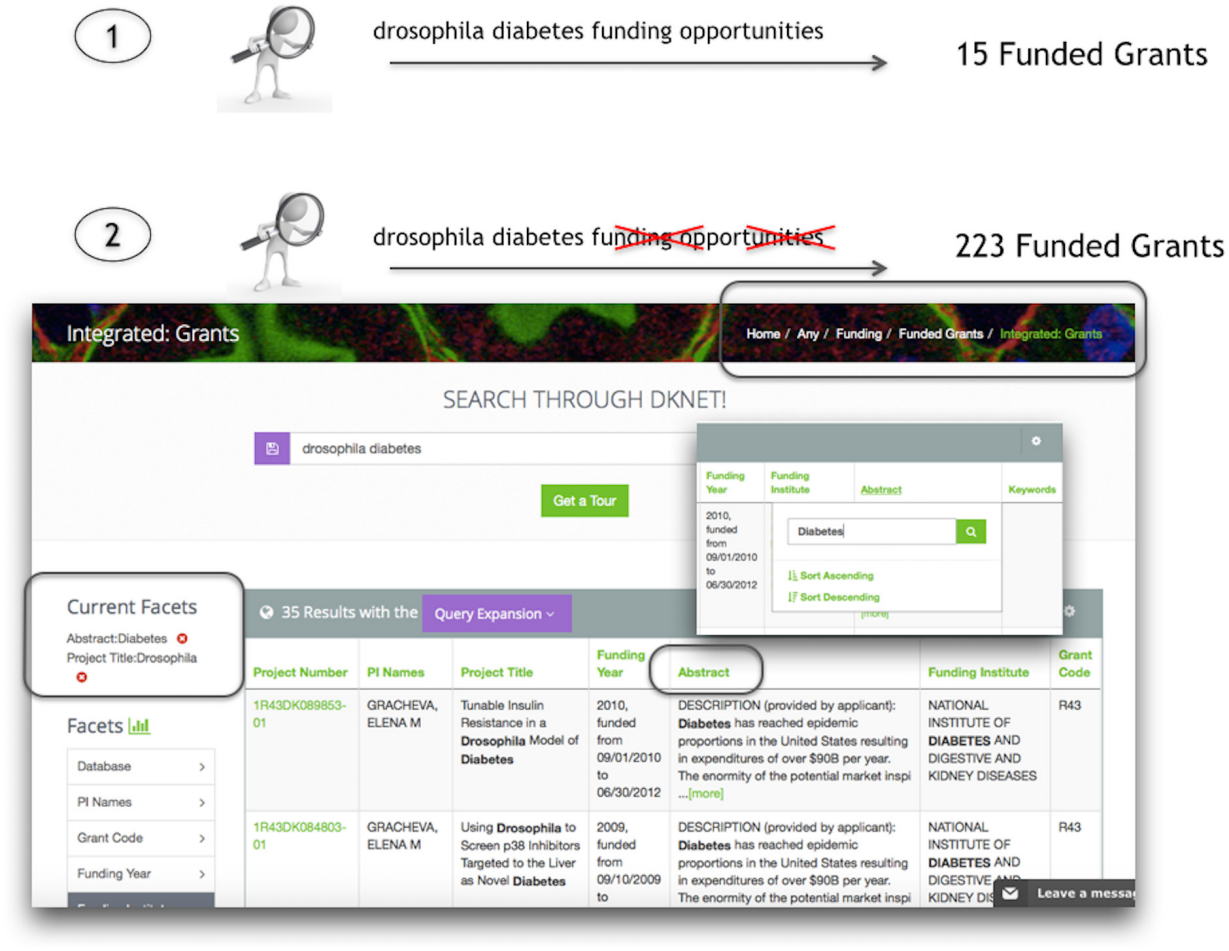
Workflow to search for funding opportunities related to drosophila and diabetes. In the first search using the terms “drosophila diabetes funding opportunities” a limited number of results are returned. Since dkNET includes a funding category and an opportunities subcategory, theses terms can be removed as search terms, therefore expanding the search and returns 223 (6/2015) records. This expanded set of results can then be further filtered in the table view.

In general, queries across data sources perform better when users start with general terms and then use the facets and filters to narrow their search. In this case, removing the terms “funding” and “opportunities” provides a larger set of query results within NIH Reporter, as these terms are no longer necessary once inside the funding data sources (Fig 7).

Although this query did not find any opportunities-not completely surprising given the specificities of the terms-information about NIH programs that have previously funded such models can be obtained through NIH Reporter. Switching to table view, the Funding Institute facet can be used to obtain a list of NIH institutes that have funded drosophila models of diabetes. Examination of the search results show that the first record Insulin-Dependent Diabetes Mellitus (Project Number 1ZIADE000423-25) is a grant about diabetes mellitus, although the term Drosophila does not appear in the project title. However, expanding the project description shows that drosophila occurs in the description: *“Further studies showed that IA-2/IA-2beta belong to an ancient gene family going back 500 million years with homologs in Drosophila and C*. *elegans*.*”* In this case, using the facets and filters can help to provide a more relevant set of search results. For example, entering “drosophila” into the “Project title” column provides a more focused set of results, as does entering “Diabetes” into the “Abstract” column (Fig 7). As described in a previous section, the general ranking algorithm that operates across all data sources is based on relevancy, not by date. However, the most recent awards can be viewed by clicking on the “sort descending” function accessed via the Funding Year column heading. Clicking on the record will take the user to the same record in the NIH Reporter Database. Here, users can explore the data source further, e.g., to find the specific program announcements that funded the research.

#### [UC2] Find animal models of diabetes induced by diet

Query: diet-induced diabetes animal model. Navigation: Community Resources > Organism. This use case can be answered by entering the above search terms and then navigating to the Organisms category under Community Resources. Selecting Organism shows 6 results from 3 sources: MMPC, DiaComp and Integrated Animal, which is a compilation of data across multiple model organism databases. Five of these results point to the C56Bl/6 mouse, as expanding to the full record indicates that it is susceptible to diet-induced obesity and Type 2 diabetes. One of the records indicates that a genetically modified mouse: Tlr5^tm1Flv^/Tlr5^tm1Flv via^ is subject to diet-induced obesity and diabetes and is available through Mouse Genome Informatics (MGI). Clicking on the name brings up the appropriate page in the MGI database.

Users may also want to explore what is available under the “More Resources” tab. When this tab is selected, the query is re-executed across the 200+ databases currently available. Note that all of the databases available via Community Resources are also in this section, although the views may be organized differently. For example, under the Phenotype category in “More Resources”, the MGI Mouse Phenotype database provides an additional 31 results, of which several show either increased or decreased susceptibility to diet-induced obesity. We should note, however, that the majority of records for this database do not explicitly mention diabetes within the phenotype description, but were returned because the mice were described in a publication in the journal “Diabetes”. If a user is specifically interested in animals that exhibit diabetes, results can be further refined by entering “diabetes” as a filter in the “Description” column. This action returns a mouse model that is described as “... protected from high-fat diet-induced diabetes,”.

#### [UC3] What markers differentiate developing ureteric bud from mesonephric duct?

Query: ureteric bud mesonephric duct. Navigation: More Resources > Microarray > GEO. For this use case, a user would likely be looking for a list of genes that differentiate these two structures. The Community Resources show that Expression is a subcategory under Data, although there are no results returned. Clicking on the “More Resources” tab shows 201 results across 13 data sources. Selecting the Type of Data facet reveals results under Expression and Microarray. In this case, further exploration reveals that the GEO database has a microarray data set GEO18260 that looks at: “Expression data for kidney progenitor tissue from rat embryo at E13: In this study we compared genes expressed in the unbudded portion of the Wolffian duct with the isolated ureteric bud to find genes novel to early kidney development. “Wolffian duct is included in the search as a synonym for mesonephric duct. Although this data set does not, on its own, give us an answer to the query, there are tools that can be used to calculate differential gene expression between two samples, e.g., the Geo2R tool provided by GEO. Users can also search for software tools through dkNET by entering search terms such as: Microarray analysis and selecting the Software category.

#### [UC 4] Can I find validated antibodies against CART?

Query: CART, Navigation: Community Resources > Materials > Antibodies. In this case, we chose the simplest query: CART, which auto-expands into cartpt and cocaine- and amphetamine-regulated transcript protein. Navigating to the Antibody Subcategory under Materials, there are >80 results returned from two sources: the Antibody Registry and the BCBC.

The interesting part of this use case is the term “validated”. Including this term in the query leads to zero results, because the term doesn’t appear anywhere in the antibody databases. Antibodies are messy reagents and the issue of validation can be a contentious one and is use-case specific, but there are criteria that people use in determining whether or not an antibody can and should be used in a given application.

In dkNET, we do have several potential criteria that would stand in proxy for the term “validated”:

1. The Community Resources provides access to NIDDK-supported centers that provide antibodies that have been carefully tested by the community. In this case, the BCBC provides characterization of antibodies against CART.
2. The Antibody Registry provides a reference field where evidence of use of a particular antibody is indicated. By clicking on the column header, users can change the sorting order to show records associated with citations. Browsing the list shows that AB_2313614 (http://antibodyregistry.org/AB_2313614), for example, recognizes mouse CART and has been used in 5 published studies. AB_2275127 (http://antibodyregistry.org/AB_2275127) recognizes human and has been cited in two publications.

Thus, in this use case, an understanding of what data sources are available and how they are structured allows a user to develop an operational definition of what a researcher might consider as validated, or at least provides a reasonable starting point for looking for antibodies that meet validation criteria.

## Discussion

### Rationale of dkNET

dkNET was designed as a discovery portal for research resources of relevance to the mission of the NIDDK. The design of dkNET balances the need for a focused set of resources specifically developed by and for the NIDDK-funded research community with the broad array and growing number of biomedical databases available. Resources available under Community Resources are largely derived from projects funded by NIDDK to provide materials, protocols and data for researchers. These resources are selected by the dkNET Steering and Advisory Committees after careful review. In contrast, databases in the More Resources categories are accessed via SciCrunch, a growing library of data sources that span biomedicine. These data sources have been ingested by various communities that work through SciCrunch to create community-based data portals.

dkNET assists users in searching and browsing distributed, heterogeneous databases by providing unified search and display. Thus, dkNET is not meant to replace the individual resources indexed, but to make them easier to find and use, much as a web search engine connects users to web pages maintained by individual organizations. Within the Community Resources section, ~20 separate sources are currently searched. Each of these has its own user interface and customized display. Although the learning curve for each resource may be relatively small for basic functions, if a user needs to quickly search across multiple databases the lack of uniformity can be a significant barrier. Thus, dkNET is a working example of a Data Discovery Index, querying across the contents of distributed and heterogeneous databases. The creation of a Data Discovery Index is one of the core objectives of the recently launched BD2K (“Big Data to Knowledge”) initiative from the US National Institutes of Health (http://bd2k.nih.gov).

The information sources searched by dkNET are all examples of dynamic databases that update their content on a regular basis. The SciCrunch platform on which dkNET is built provides a complete system for ensuring that the content of dkNET is kept up to date through dynamic updates from these databases. Because SciCrunch has implemented a system of unique identifiers assigned to each individual record, modifications to individual records can be recorded and new records added each time can be identified. Thus, in a future release of dkNET, users will be able to sign up for an alerting service to let them know when new data is available.

### Challenges in designing community data portals

The current design of dkNET was meant to balance the need for a set of focused community resources against the large number of resources available. The navigation of the community section is therefore different than that provided by the more general More Resources section in that it aligns all data and data views under a common set of categories. These categories are grown organically as new types of data enter the federation. However, at some point the categories will become too numerous for this strategy to be effective. For this reason, at some point, an overarching ontology that can group related data categories and types will need to be developed. The development of such an ontology will be aided by the large amount of data already indexed by dkNET and related.portals.

A second issue is whether all of the NIDDK community, for example, should be served by a single portal or whether several sub portals serving different NIDDK communities should be designed. SciCrunch was specifically designed to create these types of portals. Setting up a specialized portal using data that is already available can ben done in as little as a few hours. All data that is ingested by any community immediately becomes available to all communities through the More Resources tab. But as communities and data sources continue to proliferate, we will have to implement better alerting services. We currently users know how many communities are using the same data sources within their Community Resources sections, but we have not yet implemented a system for letting communities know when a new data source has been added that might be of particular interest to them.

### Effective search strategies for data

The advent of web search engines has provided a high bar for those developing effective search tools by the apparent simplicity and power of a key word search. For a growing resource like dkNET, which continually adds new types of data and new data sources, we believe that the enhanced keyword interface is the most effective way to search dkNET data sources, because it doesn’t require any a priori knowledge of the contents or structure of the system. But the power of structured data is the ability to take advantage of that structure to create very specific queries that return precise answers. dkNET balances the need for broad keyword based and structured search through the use of categories, facets and filters that allow users to progressively narrow down their search according to what is available. As the use cases provided above illustrate, the most effective approach to searching dkNET is generally to start with a fairly broad term, and then use these facets to help narrow down the search. dkNET offers a variety of options for providing overall views of what is available, e.g., through the Category Graph.

The problem of querying across dynamic databases, much like with a general web search engine, is that there is almost an unlimited set of queries for which relevant information can be found. But connecting to the best set of resources for a particular question is not entirely straightforward. It is obvious for a query with a set of conditions, e.g., UC4: *Find an antibody against CART*, that if a resource exists that matches the exact request, we should be able to connect researchers quickly, transparently, and effectively to that resource.

However, as seen with the use cases presented, e.g., UC1 and 4, more often than not, a direct answer to the question does not exist, but a set of reasonable answers that provide valuable information on the topic can be discovered. For example, in UC1, the drosophila diabetes funding use case, an experienced researcher might be able to detect a reasonable set of programs for funding fly models of diabetes from the database of funded grants, particularly if s/he knows that the NIH Reporter provides information from the funding announcements for a particular funded grant. Answering such queries requires that the user be willing to explore the dkNET portal. Because having an in depth knowledge of the types of data available and the most effective types of search strategies can increase the utility of dkNET for naïve users, we have implemented a live chat feature during certain times to aid users with their queries. However, in the future, we will be working to develop recommender systems that provide users with reasonable options for particular types of queries.

### Expanding dkNET ontologies

Ontologies are used in dkNET to enhance search across databases that use different terminologies and abbreviations. Generally, the expanded searches provide additional search results compared to single terms; however, dkNET provides for the capacity to remove synonyms and search terms that cause undue numbers of false positives. The false positives occur because dkNET does not employ strict semantic search where all resource types are mapped to the ontology terms. Rather, dkNET casts a broad search using ontology terms and provides users the opportunity to narrow down using pre-determined facets, but also using any additional terms they wish. In this way, dkNET takes advantage of ontologies but is not constrained by them, and the data within dkNET can scale independent of the knowledge layers that are added as they become available. As these are developed by the community, we will incorporate them into the SciCrunch ontology. For example, future work will include imports of the Beta Cell Genomics Ontology (https://code.google.com/p/bcgo-ontology/) for improved coverage of functional genomics data from the Beta Cell Biology Consortium and the GUDMAP Ontology (http://www.gudmap.org/Resources/Ontologies.html) for expanded coverage of anatomy terms for the genitourinary tract.

### Summary

dkNET is a successful example of the type of data discovery index envisioned to propel biomedical data science as part of the NIH’s Big Data to Knowledge (BD2K) program [1]. It provides an ideal environment for illustrating some of the opportunities and challenges associated with organizing and searching the new types of digital research objects, including dynamic databases, produced in support of biomedical research. At the same time, it establishes a model for building sustainable community-focused data portals. By building on a configurable, shared platform, SciCrunch, dkNET balances the need for providing a focused set of vetted resources directly relevant to the needs of NIDDK-supported researchers, with the need for broader search across distributed databases. As dkNET ingests new data sources, other SciCrunch communities immediately benefit and vice versa. The continual updating of data sources ensures that dkNET never grows stale but, conversely, as the amount of data and tools continues to grow, connecting researchers to the most relevant resources for their particular needs will become more of a challenge. Planned enhancements include the development of auto-alert services that let users know when relevant data is added to the system and the development of recommender systems that will connect researchers to similar datasets.
